# Spatiotemporal Profiling of Starch-Degrading Enzymes in Nong-Flavor Daqu: Molecular Markers for Quantitative Quality Evaluation

**DOI:** 10.3390/foods14183239

**Published:** 2025-09-18

**Authors:** Yijia Jiang, Yue Lu, Yanling Jin, Yi Shen, Nian Liu, Shu Bao, Kui Peng, Langfei Gan, Chaokai Wang, Yuling Zhang, Lanchai Chen, Bo Chen, Yao Xiao, Kaize He, Zhuolin Yi, Hai Zhao

**Affiliations:** 1 Agricultural Microbial Agents Key Laboratory of Sichuan Province, Chengdu Institute of Biology, Chinese Academy of Sciences, Chengdu 610213, China; jyj1122330818@163.com (Y.J.); oneluyue@163.com (Y.L.); jinyl@cib.ac.cn (Y.J.); 323086002124@stu.suse.edu.cn (Y.Z.); hekz@cib.ac.cn (K.H.); 2 Liquor Brewing Biotechnology and Application Key Laboratory of Sichuan Province, Sichuan University of Science and Engineering, Yibin 644005, China; 3 Sichuan Langjiu Co., Ltd., Luzhou 646523, China; 15228262888@163.com (Y.S.); ganlf1898@163.com (L.G.); chenbo@langjiu.cn (B.C.); 4 Sichuan Food and Fermentation Industry Research & Design Institute, Chengdu 611130, China; liunian999@163.com (N.L.); 1314520pk@163.com (K.P.); gswangchaokai@126.com (C.W.); 5 Sichuan Liquor Research Institute Co., Ltd., Chengdu 610017, China; baoshujks@outlook.com; 6 School of Food and Bioengineering, Xihua University, Chengdu 610039, China; chenlc@xhu.edu.cn; 7 Analytical and Testing Center, Sichuan University of Science and Engineering, Zigong 643000, China; xiaoy828@126.com

**Keywords:** Nong flavor Daqu, starch degradation, quality evaluation, quantitative polymerase chain reaction, enzymatic activity

## Abstract

Nong-flavor (NF) Daqu, a critical fermentation starter for traditional Baijiu, harbors diverse starch-degrading enzymes with poorly characterized functional dynamics. This study transcended traditional quality assessments by developing molecular approaches to dissect starch-hydrolyzing enzyme genes. Specific and degenerate primers targeting glucoamylase, α-amylase, and α-glucosidase genes were designed, and key genes were qualitatively identified with distinct distributions among NF Daqus and unique presences between JXL and HB Daqu. Quantitative PCR revealed six genes with elevated expression in JXL Daqu versus HB Daqu, and which peaked during late fermentation in both Daqus. Metagenomics identified greater enzymatic diversity in HB Daqu. Phylogenetic clustering confirmed evolutionary conservation (GH13/GH15/GH31 families) and specificity of core enzyme genes across both Daqus. Enzymatic assays demonstrated the dominance of saccharification over α-glucosidase activity in both Daqus, with significantly higher α-glucosidase activity in JXL than HB Daqu. Divergent starch degradation strategies emerged: JXL prioritized high enzyme expression/activity, while HB utilized broader gene abundance. Based on Pearson correlation analysis, the saccharification activity showed the highest but weak correlation with α-glucosidase gene_15963 (r = 0.26), and was also positively correlated with the expression of all other enzyme genes except one glucoamylase gene. Meanwhile, α-glucosidase activity was most strongly linked to glucoamylase gene_22243 (r = 0.76), with additional correlations with two α-glucosidase genes being observed. This establishes RNA-based biomarkers for real-time quality control. Our findings decode divergent microbial strategies (JXL: high-expression/high-activity vs. HB: high-diversity) and provide a molecular framework for optimizing starch utilization in Baijiu fermentation. This technology holds potential to enable precision-driven standardization of traditional food production, which would reduce processing waste and enhance resource efficiency.

## 1. Introduction

Chinese Baijiu, recognized alongside whisky, brandy, rum, vodka, and gin as one of the world’s six major distilled spirits, is a traditional fermented and distilled beverage unique to China [1]. Its production is characterized by solid-state fermentation and the use of a microbial starter called Qu, which fundamentally distinguishes it from other global spirits [1]. Baijiu is typically produced using sorghum, corn, rice, wheat, peas, and millet, alone or in combination. Based on their distinct sensory and aromatic profiles, baijiu is classified into twelve major aroma types, including Strong (Nong), Light (Mild), Sauce (Jiang), Rice (Mi), and others [1,2]. Among these, Nong-flavor (NF) Baijiu, one of the most fundamental and popular styles, dominates approximately 70% of the Chinese liquor market [3].

The production of Nong-flavor (strong-aroma) baijiu relies on a saccharification and fermentation starter called Daqu. NF Daqu, a medium-temperature type Daqu for NF Baijiu, is primarily crafted from crushed wheat and water. This mixture is mechanically pressed into solid, parallelepiped-shaped blocks (colloquially called “bricks”), weighing approximately 3–4 kg each [4]. The moisture content of the molded raw material is typically controlled within a range of 36–40%. These “bricks” are then transferred to a specialized fermentation facility known as a Qu room (or Qu incubation room), which is designed to regulate temperature and humidity [1,4,5]. Here, they undergo spontaneous solid-state fermentation for about 30 days, and this process is then followed by a 3–6 month drying and maturation period in a storage room [1,4,5]. The fermentation of NF Daqu progresses through four key stages: initial growth, high temperature (reaching 50–60 °C), cooling, and maturation [1,4,5]. This dynamic process facilitates the gradual and stable enrichment of microorganisms originating from both the raw materials and the environment [6].

During Daqu fermentation, microorganisms produce a diverse array of enzymes, mainly including α-amylase, glucoamylase, α-glucosidase, proteases, cellulases, tannases, xylanases and pectinases [7]. Efficient starch degradation is a critical aspect of Daqu production and relies on the synergistic actions of various enzymes. Specifically, α-amylase randomly cleaves internal glycosidic bonds within starch molecules, while glucoamylase, β-amylase and α-glucosidase progressively hydrolyze glucose units from the non-reducing ends [8,9]. Although β-amylase can be present from the raw material (wheat) [10,11,12,13] and original wheat enzymes are active in the initial stage of the Daqu fermentation process [14,15], it is seldom enriched from microbes during Daqu fermentation. The primary contributors to starch degradation are the microbial saccharifying enzymes (e.g., α-amylase, glucoamylase, and α-glucosidase), which are predominantly secreted from fungi like *Aspergillus* and *Rhizopus*, as confirmed by metagenomic and metatranscriptomic studies [7,8,16,17]. These microbial enzymes, precisely regulated by fermentation parameters of temperature, humidity, and pH, form a comprehensive hydrolytic system essential for degradation of starch and the growth of beneficial brewing microorganisms, directly influencing Baijiu quality [18].

Although recent omics-based studies have provided new directions for Daqu quality evaluation in current practical scenarios in distilleries, Daqu quality evaluation has still traditionally relied on sensory evaluations and limited physicochemical parameters [19,20], falling short in systematically analyzing the complex enzyme systems within Daqu [21,22]. Moreover, many current studies have focused on environmental factors (e.g., temperature, acidity) and their impact on fermentation properties (e.g., liquefaction, saccharification, and esterification) [23,24] or functional microorganisms responsible for secreting hydrolytic enzymes (e.g., α-amylase and glucoamylase) in Daqu [22]. While enzymes play a crucial role in linking microbial activity to metabolic outcomes, there remains limited research that integrates gene expression profiles with the enzymatic functionality across different stages of Daqu fermentation.

Quantitative PCR (qPCR) technology, capable of precisely quantifying microbial populations and enzyme gene expression, has been widely applied to detect key microbial populations in black tea [25], Daqu [26], pit mud [27,28], and zaopei [29], as well as gene expression in landfill soils [30]. Yet, research on the specific roles of core amylolytic enzymes in Daqu remains scarce. Multi-omics technologies, particularly metatranscriptomics, have provided extensive data on key enzymes in NF Daqu, revealing their functional roles within the complex enzyme system [17,31,32,33,34]. Our prior metatranscriptomic analysis highlighted starch-degrading enzymes like α-amylase, α-glucosidase, and glucoamylase, with α-amylase showing notably high expression (RPKM = 465.7) at the high-temperature fermentation stage [17]. These findings underscore the importance of these enzymes in NF Daqu’s metabolic processes and lead us to hypothesize that dynamic changes in the expression of specific amylase genes are crucial to achieving starch hydrolysis efficiency.

Building on prior metatranscriptomic data, this study employs qPCR to analyze the distribution and expression of core starch-hydrolyzing enzymes across fermentation stages and mature Daqu samples. This approach, to some extent, might improve Daqu quality evaluation, and potentially provide guidance for proper Daqu production in the future. By linking microbial activity to enzyme functionality, the research offers novel insights into Daqu’s complex enzyme systems, with significant academic and industrial implications.

## 2. Materials and Methods

### 2.1. Collection of Daqu Samples

All Daqu samples were produced with wheat as the sole raw material in different Nong-flavor (NF) distilleries in Yibin and Luzhou, Sichuan, and collected at four key fermentation stages (initial growth stage: 0–8 d, high temperature stage: 8–15 d, cooling stage: 15–23 d, and maturation stage: 23–30 d). Specifically, Daqu samples from Yibin Jinxilai distillery Co., Ltd., Yibin, China (JXL) were obtained at multiple fermentation stages (0, 3, 8, 12, 21, and 30 days) under the following temperatures: 25 °C, 30 °C, 60 °C, 61 °C, 42 °C, and 25 °C, respectively. Similarly, samples from Yibin Hanbang distillery Co., Ltd., Yibin, China (HB) were collected at different fermentation stages (0, 2, 4, 15, 23, 30 days) at temperatures of 25 °C, 32 °C, 50 °C, 60 °C, 32 °C, and 25 °C, respectively (Figure S1). In addition, mature Daqu samples, aged for more than six months, were collected from multiple Nong-flavor distilleries, including Yibin Jiulixiang distillery Co., Ltd.,Yibin, China (JLX), JXL, HB, and Luzhou Longmatan Ruihua Biological Koji Co., Ltd. (RH), Luzhou, China.

During sampling, two biological replicates were set for each stage in different distilleries. Specifically, Daqu bricks were randomly selected from each room, and five sampling points were taken from each brick. The collected samples were ground into small pieces and immediately frozen in liquid nitrogen. The frozen samples were then transferred to 50 mL RNase free centrifuge tubes (430828, Corning, NY, USA) and maintained on dry ice. All samples were transported to Chengdu Institute of Biology, Chinese Academy of Sciences on the same day of collection and stored at −80 °C until further processing. For all subsequent experiments, three technical replicates were performed.

### 2.2. Design of Specific and Degenerate Primers for Starch Hydrolase Genes in NF Daqu

Previous metatranscriptomic analysis identified starch-degrading glycoside hydrolase (GH) genes (GH15, GH13, GH31), along with their quantitative abundance and expression profiles (Table S1) [17]. Gene sequences with high expression levels (RPKM > 10.0) were selected as templates for primer design (Table S2). Specific primers were designed using SnapGene software (Version 4.2, GSL Biotech, San Diego, CA, USA) and Primer Premier 5.0 software (PREMIER Biosoft, Palo Alto, CA, USA) based on the selected templates, and their quality was evaluated using Oligo Calc.

To expand the range of the detection of starch hydrolase genes, the highly expressed GH15, GH13, and GH31 genes were further used as templates to retrieve homologous sequences via BLAST+ 2.15.0 search on the NCBI database with alignment parameters set (E < 1 × 10^−5^), and gene sequences with nucleotide similarity ranging from 60% to 100% were selected as target sequences. Multiple sequence alignment was performed using MEGA 11 and DNAMAN to identify conserved and variable regions; thereafter, degenerate primers were designed to introduce variable sites at conserved regions, which thereby increased the likelihood of capturing functional variants. The quality of the degenerate primers was subsequently assessed using Oligo Calc (available at https://cail.cn/biotool/oligo/index) (accessed on 5 June 2023).

### 2.3. Total DNA Extraction, Amplification, and Sequencing of Starch Hydrolase Genes

Total microbial DNA from Daqu samples was extracted using the E.Z.N.A. Soil DNA Kit (Omega Bio-Tek, Norcross, GA, USA) and subsequently used as a template for the amplification of starch hydrolase genes. Amplification was performed using both specific and degenerate primers listed in Table 1. Approximately 20 ng of template DNA was added to 2× Rapid Taq Master Mix (Novizan Biotech Co., Ltd., Nanjing, China) and subjected to PCR following standard protocols.

The amplified products were purified using a universal DNA purification and recovery kit with spin columns (Cat. No. DP214, Tiangen Biotech Co., Ltd., Beijing, China) and ligated with the T-vector cloning kit (Cat. No. B52221, Sangon Biotech Co., Ltd., Shanghai, China) accordingly. The ligation products were then transformed into *Escherichia coli* DH5α competent cells (TransGen Biotech Co., Ltd., Beijing, China). Three recombinant clones were selected via blue-white screening, verified by colony PCR, and sequenced at Sangon Biotech Co., Ltd., Shanghai, China.

Following sequencing, plasmid sequences were removed, and the resulting sequences were analyzed for homology via BLAST tool (2.14.0.) on NCBI. Taxonomic information was retrieved to determine the species origin of the sequences.

### 2.4. RNA Extraction, cDNA Synthesis, and RT-qPCR Analysis

Total RNA was extracted from Daqu samples using a borate buffer solution, purified by the RNeasy Midi Kit (Qiagen, Cat. No. 75142, Venlo, The Netherlands), and treated with DNase I (Fermentas, Waltham, MA, USA) to remove genomic DNA contamination as described previously [17]. The purity, concentration, and integrity of the RNA were assessed using an Agilent 2100 Bioanalyzer (Agilent, Santa Clara, CA, USA), and RNA integrity was further analyzed by agarose gel electrophoresis. Possible genomic DNA contamination was detected by performing conventional PCR reaction with the purified Daqu RNA as template.

Reverse transcription was carried out using purified Daqu RNA as template with the HiScript III All-in-one RT SuperMix Perfect for qPCR kit (Novizan Biotech Co., Ltd., Nanjing, China). The reverse transcription reaction was performed in a 5 μL system under the following conditions: 50 °C for 15 min and 85 °C for 5 s, according to the manufacturer’s instructions. The synthesized cDNA was stored at −20 °C for subsequent analysis.

Primers for RT-qPCR analysis were designed using Primer Premier 5.0 (Table 2). Specificity was verified by DNA-based PCR under the same amplification conditions as described earlier. Reactions were performed in a 10 μL system containing cDNA templates and the Taq Pro Universal SYBR qPCR Master Mix kit (Novizan Biotech Co., Ltd., Nanjing, China). Furthermore, a set of experimental groups without reverse transcriptase (RT enzyme) were established, and the thermal cycling protocol included the following:

Initial denaturation: 95 °C for 30 s;

Amplification: 40 cycles of 95 °C for 10 s and 60 °C for 30 s;

Melting curve analysis: cooling to 65 °C, followed by denaturation at 95 °C for 5 s.

The cycle threshold (Ct) value, representing the cycle number at which fluorescence exceeded background levels, was recorded, and the relative gene expression was calculated by the 2^−ΔΔCt^ method, after normalization with internal reference gene *gapdh*.

### 2.5. Diversity Analysis of Carbohydrate-Active Enzymes

NF Daqu samples from the HB and JXL distilleries were subjected to metagenomic sequencing on the Illumina Hiseq™ platform at Sangon Biotech Co., Ltd., Shanghai, China. A total of 12 samples yielded an average of 6.0 Gb of raw paired-end (150 bp) sequencing data per sample. After quality control (adapter removal, quality trimming, and filtering) using Fastp (v0.36), a total of 33.5 Gb of high-quality clean data was obtained.

The clean reads from all samples were co-assembled using Megahit (v1.2.9). Unmapped reads were subsequently extracted and reassembled with SPAdes (v3.13) to improve the recovery of low-abundance genomes. Contigs shorter than 500 bp were discarded. The final assembly resulted in 1,891,979 contigs with an N50 of 982 bp. Metagenome-assembled genomes (MAGs) were reconstructed using the MetaWRAP (v1.3.2) pipeline, including binning, refinement, quantification, reassembly, and taxonomic classification. Only bins with >70% completeness and <10% contamination were retained for downstream analysis.

The HMMER3 tool was employed to search for and align gene-encoded protein sequences within the Carbohydrate-Active Enzymes (CAZy) database (http://www.cazy.org/) (accessed on 29 January 2024), with the aim of identifying and annotating enzymes associated with carbohydrate metabolism. To ensure the accuracy and biological significance of the results, a stringent statistical threshold (E-value < 1 × 10^−5^) was applied during the search process. Based on the annotations, a comprehensive statistical analysis was conducted to assess the distribution density of enzymes across various functional categories within the CAZy database.

### 2.6. Determination of Saccharification and α-Glucosidase Activities in NF Daqu

The crude enzyme extraction from Daqu was performed according to a previously described method [17,35,36] with minor modification. Briefly, triplicate 10 g portions of each NF Daqu sample were mixed with 40 mL of ultrapure water and incubated at 30 °C for 1 h. The mixture was then centrifuged at 5500 rpm for 10 min, and the supernatant was collected as the crude enzyme extract for subsequent assays.

The saccharification activity was assessed according to revised protocols [20,36,37,38,39,40,41,42] with soluble starch as substrate. The crude enzyme extract was diluted at a 1:4 (*v*:*v*) in a series of buffers: citric acid buffers (pH 3.0–6.0), phosphoric acid buffers (pH 6.0–8.0), or Tris-HCl buffers (pH 8.0–9.0). The enzymatic reaction was initiated by adding 10 mg/mL soluble starch as the substrate and was carried out under varying temperature (40 °C to 90 °C) and pH conditions (pH 3.0–9.0) for 30 min. All experiments were performed in triplicate. The amount of reducing sugar released was quantified using the 4-*p*-hydroxybenzoyl hydrazide (*p*HBAH, Sigma-Aldrich, St. Louis, MO, USA) method [37,39,43]. Glucose was used as a standard, and one unit of saccharification activity was defined as the amount of enzyme required to hydrolyze the starch to produce 1 μmol of glucose equivalents per minute under the specific conditions.

The extraction method for α-glucosidase was identical to that used for the saccharification activity. The activity profile of α-glucosidase was determined using *p*-nitrophenyl α-D-glucopyranoside (*p*NPG) as the substrate, following a well-characterized procedure [44,45,46,47]. Similarly, the reaction profile was performed under varying pH (pH 3.0–9.0) and temperature (40–90 °C) for 60 min. Each set of experiments were conducted in triplicate along with one blank test where no substrate was added. The release of *p*-nitrophenol (*p*NP) was measured by monitoring the absorbance at 410 nm using a microplate reader (Shanghai Flash Spectrum Biotechnology Co., Ltd., Shanghai, China). The activity of α-glucosidase was calculated based on the rate of *p*-nitrophenol (*p*NP) released, which was determined by comparing the results against standard *p*NP calibration curves established in different pH buffer solutions.

### 2.7. Correlation Analysis of Carbohydrate-Active Enzymes

Pearson correlation analysis was employed to identify key starch hydrolase genes and their corresponding detection primers in NF Daqu, with the statistical significance threshold set at *p* < 0.05. The relationship between gene expression levels and enzyme activities was systematically evaluated to determine genes with significant contributions to starch hydrolysis. Specifically, correlation calculations were performed using Python software (version 3.0) for data preprocessing and preliminary analysis. Subsequently, Origin software (version 2021) was utilized to generate high-quality visualizations.

## 3. Results

### 3.1. Preliminary Analysis of DNA-Based Amplification of Target Starch Hydrolase Genes and Their Distribution in Different NF Daqu Samples

Based on previous metatranscriptome analyses of NF Daqu from the HLM distillery [17], highly expressed genes associated with starch degradation were selected that encode glucoamylase, α-amylase, and α-glucosidase enzymes belonging to the GH15, GH13, and GH31 families, respectively. The specific primers GH15-1, GH15-4, GH13-1, GH13-3, GH31-1, and GH31-3, along with the degenerate primers 22243, 19398, 17723, 31847, 22983, 15963, and 17558, were designed to amplify these highly expressed genes and their homologous sequences, with Daqu DNA being used as a template.

Gel electrophoresis analysis revealed that most Daqu samples exhibited bands corresponding to the expected target DNA fragments (Figure S2). However, exceptions were observed in certain samples: the N2 sample (almost all primers), the N3 sample (specific primers GH13-1 and GH13-3), and the HB and JXL Daqu samples (specific primer GH31-3 and degenerate primers 31847, 22983, and 15963) (Figure S2). The amplified DNA fragments were subsequently purified, cloned, and sequenced.

As shown in Table S3, most of the amplified fragments were annotated as glucoamylase, α-amylase, and α-glucosidase genes, predominantly originating from fungal genera such as *Aspergillus*, *Penicillium*, *Thermomyces*, *Rasamsonia*, and *Paecilomyces*. Specifically, among the specific primers, the GH15-1 primer amplified four glucoamylase genes with nucleotide sequence similarities ranging from 41.3% to 60.1%, while the GH15-4 primer amplified six glucoamylase genes with similarities ranging from 48.0% to 73.3%. In the GH13 family, the GH13-3 primer amplified three α-amylase genes, whereas GH13-1 amplified one α-amylase gene. Similarly, in the GH31 family, both GH31-1 and GH31-3 amplified one α-glucosidase gene each, exhibiting 100% nucleotide similarity. These findings suggest that specific primers not only amplified highly similar target genes but also captured enzyme genes with varying sequence similarities (from 41.3% to 100%). Although the degenerate primers 22243, 17723, 22983, and 31847 amplified fewer enzyme genes (1–2 per primer), the obtained sequences displayed a wide nucleotide similarity range of 38.7% to 100%, indicating the broad detection capability of these primers.

**Figure 1:**
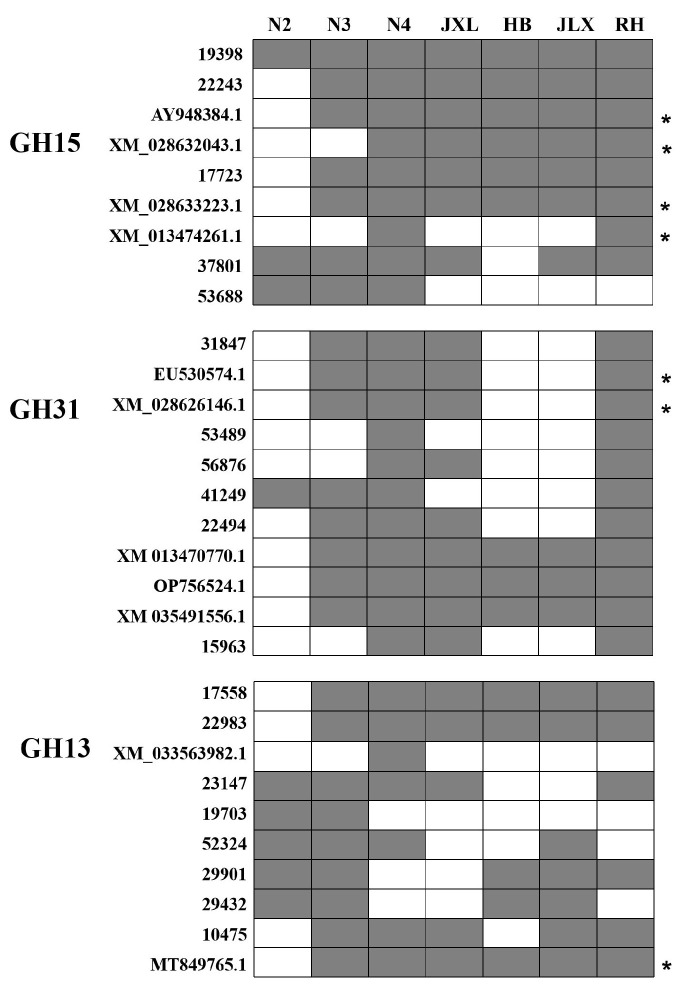
The distribution of starch hydrolases genes in different NF Daqu samples. The vertical axis represents different starch hydrolase genes. The horizontal axis represents different NF Daqu samples. The fermentation Daqu samples were collected from Yibin HLM distilleries (N2:5 d; N3:15 d; N4:30 d), and mature Daqu samples were collected from different NF distilleries (HB, JLX, JXL and RH) in Yibin and Luzhou Cities. The asterisk indicated novel genes identified with low similarities (<80%) to templates.

According to DNA-based amplification and sequencing of starch hydrolase target genes, a comprehensive analysis was conducted to elucidate their distribution across different NF Daqu samples. In addition to the genes previously identified through metatranscriptomics, several novel genes were also detected, including AY948384.1 (*Thermomyces lanuginosus*), XM_028632043.1 (*Paecilomyces variotii*), XM_028633223.1 (*Paecilomyces variotii*), XM_013474261.1 (*Rasamsonia emersonii*), EU530574.1 (*Thermomyces lanuginosus*), and XM_028626146.1 (*Paecilomyces variotii*) (Figure 1). In the GH15 family, gene 19398 was consistently detected throughout the fermentation and maturation stages, while genes 22243, AY948384.1, 17723, and XM_028633223.1 were identified in high-temperature fermentation sample N3 and mature Daqu samples. In contrast, gene 53688 was exclusively detected during the Daqu fermentation stages (N2, N3, and N4). For the GH13 family, genes 17558, 22983, and MT849765.1 were consistently identified throughout the fermentation and maturation stages, except in N2. In the GH31 family, genes XM_013470770.1, OP756524.1, and XM_035491556.1 were detected across all fermentation and maturation stages, except in N2. Additional genes, including 31847, EU53054.1, XM_028626146.1, and 22494, were observed during the fermentation stages (N3, N4) and in mature Daqu samples from the JXL and RH distilleries. Interestingly, several genes were absent in mature Daqu samples from the HB and JLX distilleries, including 31847, EU530574.1, XM_028626146, 56876, 22494, 15963, and 23147. Taking into account their close distance (within 4 Km) allows the inference that there may be potential similarities in the Daqu-making processes and environment at these distilleries. In conclusion, the genes 19398, 22243, 17723, 17558, 22983, 31847, and 15963 were identified as key amylolytic enzyme genes in NF Daqu.

### 3.2. Distribution of Key Starch Hydrolase Genes During the Daqu-Making Process in Two Representative NF Daqus by DNA-Based Amplification

The identified key starch hydrolase genes were selected for further quantitative verification during the fermentation process of NF Daqu samples from the HB and JXL distilleries. Corresponding detection primers were developed, including the specific primers GH15-4, GH13-3, and GH31-1 and the degenerate primers 19398, 22243, 17723, 17558, 22983, 31847, and 15963.

**Figure 2:**
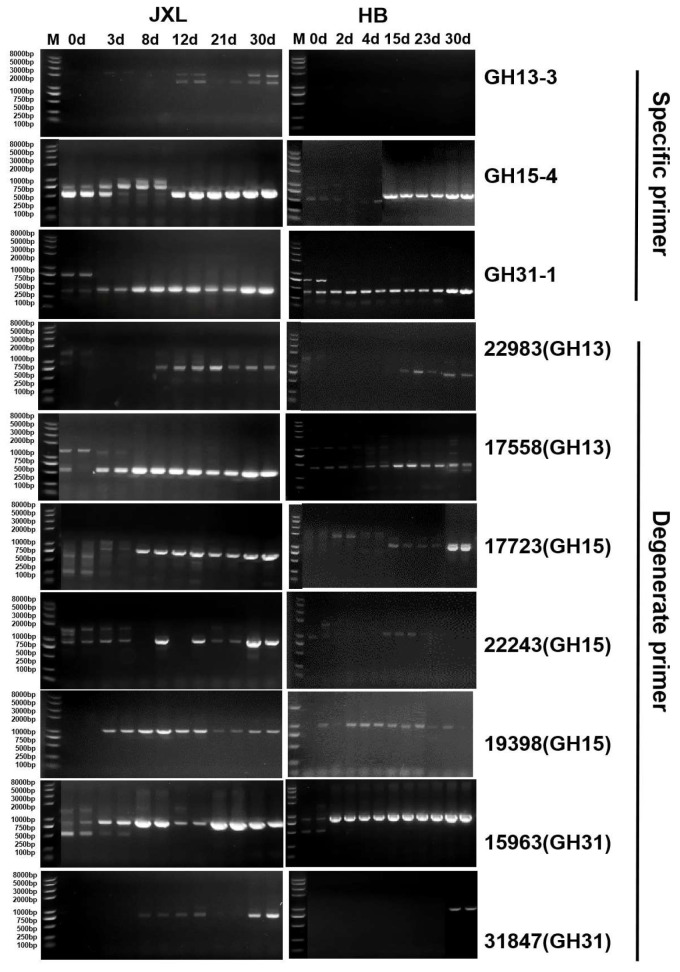
DNA-level amplification results of JXL and HB Daqu samples during Daqu-making fermentation process. The Daqu samples were collected on different fermentation days from JXL (day 0, 3, 8, 12, 21, and 30) and HB (day 0, 2, 4, 15, 23, and 30) distilleries, respectively.

In the JXL distillery, glucoamylase genes from the GH15 family, detected using primers 22243 and 19398, were absent in the early fermentation (0 to 3 days) and stably present throughout the later stages (Figure 2). Similarly, the glucoamylase gene detected by the GH15-4 primer showed enrichment after 12 days of fermentation and remained stable during the middle and mature stages. For the GH13 family, the α-amylase gene detected by primer 17558 was consistently present throughout the entire fermentation process, whereas the gene detected by primer 22983 gradually accumulated after 12 days of fermentation and stabilized in the subsequent stages. However, the α-amylase gene detected by the GH13-3 primer was absent throughout the fermentation process. In the GH31 family, α-glucosidase genes detected using primers 15963 and GH31-1 were absent only at the initial stage (0 days) and were rapidly enriched and stably maintained during the middle fermentation and mature stages. The target gene detected by primer 31847 showed enrichment after 8 days of fermentation and remained present in the subsequent stages.

In HB Daqu, the distribution of starch-hydrolyzing enzyme genes exhibited significant differences compared to JXL Daqu. The GH15 family genes detected by primer 17723 were absent in the early fermentation stages (2 to 4 days) but enriched after 15 days and remained stable thereafter, while genes detected by primer 22243 were only clearly detected in the late fermentation stages (15 to 23 days) (Figure 2). In contrast, the genes detected by primers 19398 and GH15-4 were consistently present throughout the entire Daqu-making process, from the initial fermentation stage (2 days) to the mature stage (30 days). For the GH13 family, the α-amylase genes detected by primer GH13-3 were absent during all fermentation stages. Primer 22983 failed to amplify the target gene in the early fermentation stages (0 to 4 days) but successfully detected it in all later stages. Similar to the findings in JXL Daqu, the α-amylase gene detected by primer 17558 was stably present throughout the fermentation process. In the GH31 family, the primer 31847 successfully amplified the target gene only in the mature stage (30 days), whereas primers 15963 and GH31-1 consistently detected α-glucosidase genes throughout the entire fermentation process.

Based on the distribution patterns of starch hydrolase genes during the fermentation processes of JXL and HB Daqu, two primers from each GH family were selected for further quantitative analysis. These primers include 17558 and 22983 (GH13), 22243 and 19398 (GH15), and 15963 and 31847 (GH31), as they consistently amplified target genes in most fermentation stages.

### 3.3. Fluorescence Quantitative Analysis of the Relative Expression Levels of Starch Hydrolase Genes During the NF Daqu-Making Process

Specific primers (q17558, q22983, q22243, q19398, q15963, q31847) were designed for the RT-qPCR of key starch hydrolase genes, and all of them could successfully amplify a single band from most of the Daqu-making stages when Daqu DNA was used as a template, which definitely indicated their specificities (Figure S3). The high quality of the purified Daqu RNA was confirmed by both an RNA Integrity Number (RIN) > 7 and the presence of two distinct ribosomal RNA bands in agarose gel electrophoresis. No bands were obtained when conventional PCR was performed using Daqu RNA as the template, which indicates the absence of genomic DNA contamination. Furthermore, when using cDNA as the template, melting curve analysis showed that all of the primers consistently displayed a single peak without any miscellaneous peaks in multiple tests and replicate experiments, which indirectly indicates normal amplification efficiency (Figure S4). This result further confirmed the uniqueness and specificity of the amplification products.

**Figure 3:**
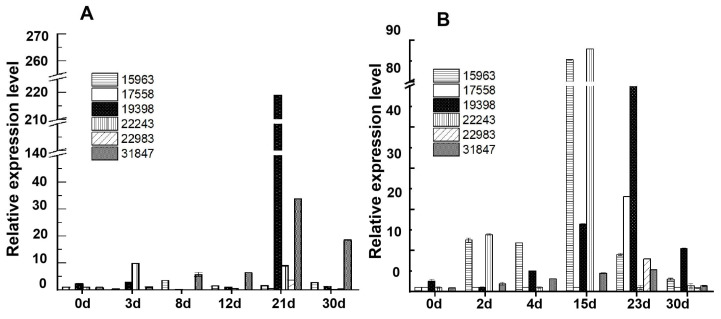
RT-qPCR analysis of starch hydrolase genes in JXL and HB Daqu samples. Transcript abundances were normalized to the *gapdh* gene as an internal reference, and the vertical axis represents relative gene expression level (2^−ΔΔCt^). The horizontal axis represents Daqu samples collected at different fermentation days from JXL distillery (**A**) and HB distillery (**B**), respectively. Data are presented as the mean ± standard deviation (error bars) of three biological replicates.

When analyzed by qPCR, the relative expression level (2^−ΔΔCt^) of glucoamylase gene 19398 exhibited an increase from the initial to middle fermentation stages (0 day to 21 days) and a decrease at the end of the fermentation stage in JXL distilleries, with the highest value (2^−ΔΔCt^ = 219.0) being observed after fermentation for 21 days (Figure 3). The relative expression level of 22243 was very low from the beginning (0 day) to the end (30 days) of fermentation (<6.9), whereas, in HB distilleries, glucoamylase genes of 19398 and 22243 all showed lower values than those in JXL and exhibited the highest values of 53.0 and 88.0 after fermentation for 23 days and 15 days, respectively.

For α-glucosidase genes (31847 and 15963) in JXL distilleries, the relative expression level of 31847 showed pretty low values (<6.0) from initial stage (0 day) to mid-fermentation (12 day), and reached its peak value (33.8) after fermentation for 21 days. Meanwhile, 15963 exhibited serial low values (<10.0) in all fermentation stages. However, in HB distilleries, 31847 showed pretty low expression levels (<5.0) throughout the making process, while 15963 exhibited higher values than 31847 and reached its highest value (85.0) after fermentation for 15 days.

For α-amylase genes in JXL distilleries, the relative expression levels of 22983 and 17558 were extremely low (<10.0) from early fermentation (0 day) to the mature Daqu stage. In HB distilleries, the expression levels of 22983 and 17558 were also very low, but a litter higher than those of JXL, and both reached their peaks in the late fermentation stage (23 days), which were 8.0 and 23.1, respectively.

Therefore, most of the enzyme genes had higher expression levels in JXL distilleries compared to the HB distilleries, with high-expression genes mainly being enriched in the later fermentation stages. For example, glucoamylase gene 19398 peaked at 21 days in JXL and 23 days in HB, gene 22243 reached its peak at 15 days in HB, and α-amylase genes 22983 and 17558 showed the highest expression levels at 23 days in HB. These differences suggested notable variations in enzyme content between Daqu samples from different distilleries, indicating that the expression levels of enzyme genes could provide a certain reference for detecting and differentiating Daqu fermentation stages, while also highlighting the importance of these enzyme genes in shaping Daqu characteristics. Additionally, certain genes, such as 22243 and 19398, exhibited relatively high expression levels in the mature stage, which might play a key role in determining the quality of mature Daqu. Overall, qPCR revealed dynamic expression patterns of key enzyme genes (e.g., α-glucosidase, α-amylase, and glucoamylase) throughout Daqu production, providing foundational insights for further development of fermentation monitoring tools.

### 3.4. Effects of Temperature and pH on the Activities of Saccharification and α-Glucosidases During the Daqu-Making Process in JXL and HB Distilleries

This study evaluated the effects of temperature (40–85 °C) and pH (pH 3.0–pH 9.0) on the activities of saccharification and α-glucosidases of Daqu samples in two distilleries, JXL and HB. It should be noted that, since NF Daqu constitutes a complex enzyme system, the activity of saccharification represents the overall starch-degrading activity, while the activity of α- glucosidase indicates the overall exo-acting activity on hydrolyzing starch under varying environmental conditions.

**Figure 4:**
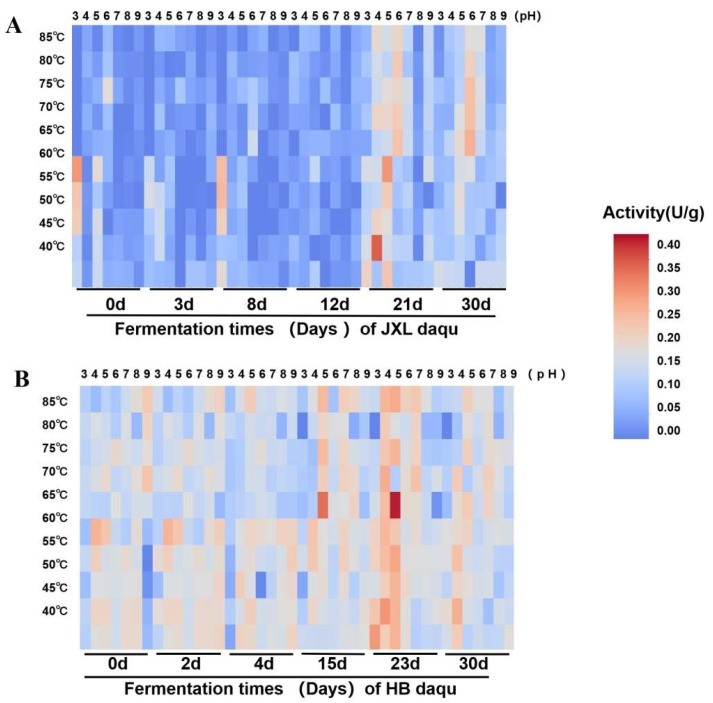
Enzymatic characteristics of the saccharification during fermentation process of JXL and HB Daqu. (**A**) The saccharification activities in the fermentation process of JXL Daqu; (**B**) The saccharification activities in the fermentation process of HB Daqu. Data were presented as the mean ± standard deviation (error bars) of three biological replicates.

For saccharification activities in a JXL distillery, the highest activity (2.61 U/g) was found at 45 °C and pH4 after fermentation for 21 days, and the mature Daqu (30 day) showed higher activities under pH 5.0–7.0 and 60–70°C (Figure 4A). Interestingly, several distinct peaks could be clearly identified in fermentation process, e.g., 21 day, suggesting the presence of multiple saccharification enzymes with different characteristics within the complex system of Daqu. Meanwhile, in the HB distillery, the saccharification enzymes were enriched with high activities in the late fermentation stages (15 and 23 day) at 65 °C and pH 5.0, and the highest activity (2.76 U/g) after fermentation for 23 days, which was the same as that in JXL Daqu (Figure 4B). Similarly, several peaks were also observed from most of the fermentation stages. In addition, low activities (0.23 U/g) were also clearly detected at the beginning stage (0 day) of both distilleries.

Throughout the fermentation stages in the JXL distillery, α-glucosidases only showed pretty low activities (<0.53 U/g) at pH 7.0 and temperature ranges of 40 °C to 60 °C (Figure S5A). In contrast, almost no activity of α-glucosidase was detected in the Daqu samples in the fermentation stages at the HB distillery, and extremely low activities (<0.51 U/g) were detected in mature Daqu (30 days) under conditions of pH 7.0 and 40 °C (Figure S5B) which was significantly lower than the peak activities in mature Daqu (30 days) from JXL.

In other words, both the JXL and HB distilleries were enriched with complex starch-degrading enzymes during the Daqu-making process, but enzyme activities and succession patterns varied. The saccharification activity peaked around days 21–23, with slightly higher values in HB than JXL, though both decreased significantly in the maturation phase. Both distilleries were also enriched with a substantial number of thermostable saccharification enzymes. For α-glucosidases, JXL’s activity gradually increased from days 12 to 30, while HB was only observed with activity in the maturation phase, which was lower than that in JXL.

### 3.5. Metagenomic Analysis of Starch-Hydrolyzing Enzymes in NF Daqu

For each Daqu sample, clean reads were obtained with high percentages, ranging from 89.3% to 97.9%. The average lengths of all assembled contigs and predicted genes were 1013.6 bp and 433.2 bp, respectively. Based on alignment against the CAZy database, all genes encoding enzymes involved in starch degradation—namely α-amylases (GH13 family), glucoamylases (GH15 family), and α-glucosidases (GH31 family)—were selected for further analysis.

**Figure 5:**
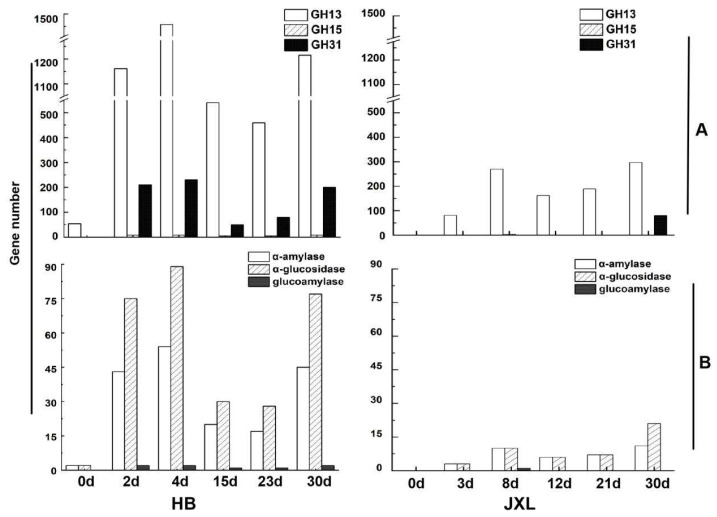
Metagenomic analysis of starch hydrolases during fermentation stages of JXL and HB Daqus. (**A**): The gene count of glycoside hydrolase family; (**B**): the gene count of starch-degrading enzymes (α-amylase, glucoamylase and α-glucosidase).

As illustrated in Figure 5A, the GH13 family was identified as the most abundant glycoside hydrolase family, as it exhibited the highest gene count throughout the fermentation process in both distilleries, followed by the GH31 and GH15 families. Specifically, in HB Daqu, the number of GH13 family genes increased rapidly to 1458 by day 4, then gradually decreased and stabilized at approximately 1215 in mature Daqu (30 days) (Figure 5A). A similar trend was observed in JXL Daqu, where the GH13 gene count peaked at day 8 and remained relatively high in the maturation stage.

In contrast, the gene count for the GH31 family remained relatively low in both distilleries, with HB exhibiting slightly higher levels than JXL. The highest GH31 gene count in HB Daqu was observed at day 4 (230 genes), while, in JXL, the gene count peaked at day 30 (80 genes). The GH15 family genes were present in significantly lower numbers, with fewer than 10 genes being detected during the late fermentation and maturation stages (HB: 23-day and 30-day Daqu; JXL: 30-day Daqu). Notably, the distribution of GH31 and GH15 genes differed significantly between the two distilleries.

Additionally, α-amylase and α-glucosidase were the predominant starch-degrading enzymes in both distilleries, with significantly higher gene counts compared to glucoamylase (Figure 5B). Similar trends to those seen for GH13 and GH31 family members were observed for the α-amylase and α-glucosidase gene counts, with peak values occurring during the initial fermentation stage and the levels remaining relatively high levels in mature Daqu. Furthermore, the total gene counts of annotated α-amylase, α-glucosidase, and glucoamylase were lower than the overall gene counts of the GH13, GH31, and GH15 families, which suggests the potential presence of a substantial proportion of hydrolases that exhibit functional diversity beyond current annotations within those families of NF Daqu.

To further elucidate the diversity of starch-hydrolyzing enzymes, gene sequences obtained through metagenomic sequencing across different fermentation stages were subjected to phylogenetic clustering analysis alongside key enzyme genes that were previously detected. As shown in Figures S6 and S7, each 17723, 22243, and 19398 gene in the GH15 family clustered separately. In the GH31 family, genes 15963 and 31847 also clustered independently. Similarly, each 22983 and 17558 gene from the GH13 family formed its own distinct clusters.

### 3.6. Correlation Analysis of Starch Hydrolyzing Activity and Gene Expression in JXL and HB Daqu

**Figure 6:**
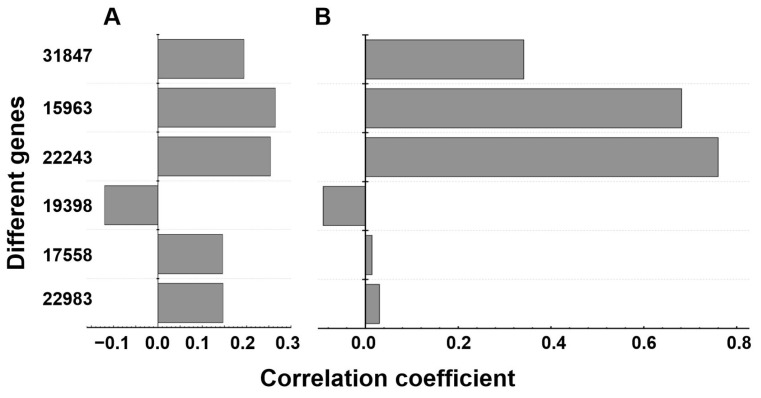
Correlation between activities (saccharification and α-glucosidase) and gene expression in JXL and HB Daqus. (**A**): Correlation between saccharification activity and gene expression; (**B**): Correlation between α-glucosidase activity and gene expression.

As illustrated in Figure 6, most starch-degrading enzyme genes exhibited a positive correlation between their expression levels and the saccharification activity. Among them, gene_15963 demonstrated the highest positive correlation, although the strength of this association was weak (r = 0.26). Conversely, gene_19398 showed a negative correlation between its expression level and the saccharification activity (r = −0.12).

For α-glucosidase activity, the expression levels of gene_31847 (r = 0.34), gene_15963 (r = 0.69), and gene_22243 (r = 0.76) exhibited strong positive correlations. In contrast, gene_19398 displayed a slight negative correlation with α-glucosidase activity (r = −0.09). The correlation values for gene_17558 and gene_22983 were low. Alternatively, the correlation values for gene_17558 and gene_22983 were statistically insignificant.

Thus, the saccharification activities are primarily regulated by gene_22983, gene_17558, gene_15963, gene_22243, and gene_31847. Meanwhile α-glucosidase activities are predominantly associated with gene_31847, gene_15963, and gene_22243. Overall, the results provide valuable insights into the molecular mechanisms underlying starch hydrolysis in Daqu production and highlight key genes for enzyme activity regulation. Understanding these correlations may facilitate the optimization of fermentation conditions to enhance Daqu quality and enzymatic efficiency.

## 4. Discussion

NF Daqu, a key saccharification and fermentation agent in Chinese NF liquor production, harbors diverse functional enzymes critical for starch hydrolysis. Metagenomic and metatranscriptomic analyses have identified active starch-degrading enzymes (GH15, GH13, GH31 families) as central players in this process [2,8,17,48]. Despite functional similarities, enzyme profiles vary across distilleries due to open fermentation conditions. Even within the same distillery, distinct α-amylase distributions between high- and low-quality Daqus highlight the impact of production processes and environmental factors [49]. RT-qPCR enables precise quantification of RNA expression levels [50]. When combined with DNA-based PCR, RT-qPCR serves as a robust tool for biological studies. These studies include Daqu, soil, air, and pit mud analysis. However, the expression patterns of starch-degrading enzyme genes across different distilleries and fermentation stages remain poorly characterized, and this poor characterization would further limit industrial optimization efforts.

Building on prior metatranscriptomic data [17], this study designed specific and degenerate primers to systematically analyze starch hydrolase genes (GH15, GH13, GH31) across distilleries and fermentation stages. Target bands amplified from Daqu samples were identified as glucoamylase, α-amylase, and α-glucosidase genes, primarily from the *Aspergillus*, *Penicillium*, *Thermomyces*, *Rasamsonia*, and *Paecilomyces* genera. This result was consistent with previous reports on the microbial origins of these enzymes [38,51,52,53,54]. All of these microbes, especially *Aspergillus* and *Thermomyces,* were reported as the domain communities in NF Daqu [1,2,24,55,56,57], which might indicate the pivotal roles of those enzymes that were detected in NF Daqu. The primers also detected homologous genes, with nucleotide sequence similarities ranging from 38.7% to 100% across enzyme families, demonstrating their broad detection efficiency. This approach enables rapid assessment of functional enzyme distribution in Daqu, akin to methods used in hot spring environments [58].

Numerous starch-degrading enzyme genes, including GH15 (19398, 22243, GH15-1/-4), GH13 (17558, 22983), and GH31 (31847, GH31-1), showed conserved enrichment across mature NF Daqus, and most of them were identified as being from *Aspergillus, Paecilomyces*, and *Thermomyces* (Table S3), the dominant fungal genera in NF Daqu. Their stabilities possibly indicated skill-dependent distribution patterns that reflected their functional importance, which, to some extent, was consistent with previous findings that the enzymatic properties of NF Daqu across different regions were influenced by their key fungal composition, e.g., *Aspergillus* and *Thermomyces* [36]. Additionally, some genes showed differential presence across mature NF Daqus, which suggests environment-dependent distribution patterns and emphasizes their specific functions. This is similar to the variations observed in high-temperature Daqus from Qingzhou and Xiangyang [59]. Even within the same distillery, variations in production location led to three distinct types of high-temperature Daqu (white, yellow, and black), which highlights micro-environmental impacts [20,60].

Functional gene profiling revealed that HB Daqu had the closest affinity to JLX, then JXL, one possible reason for which was shared processes and geographically proximal (within 20 km) environments that drive microbial-enzyme network convergence [2]. This spatial–functional correlation underscores starch-degrading enzymes as core determinants of NF Daqu quality. HB and JXL were thus selected for further comparative analysis to dissect process-driven versus location-specific regulatory mechanisms.

Ultimately, we identified key starch hydrolase genes in Daqu production, and their distributions were further compared between the HB and JLX distilleries. Core enzyme systems (GH31-1, 17558, 19398, 15963) were identified to consistently present throughout fermentation in both distilleries. Auxiliary enzyme systems, such as 31847, 22983, GH15-4, and 17723, were differentially enriched at specific stages in the fermentation process. Additionally, distinct enzymes like 22243 and GH13-3 exhibited unique distributions across the distilleries. These variations may stem from differences in fermentation environments and microbial communities across distilleries [61]. Our findings offer insights into the distribution and functional roles of key starch hydrolase genes, as well as potential improvements in Daqu quality evaluation.

qPCR analysis using specific primers (q22243, q19398, q31847, q15963, q22983, q17558) quantified the starch hydrolase gene expression in JXL and HB Daqu samples. The primer specificity was validated by DNA/cDNA-level amplification [62], with initial sample exceptions, likely due to low expression or sequence diversity [63]. Aligning with metatranscriptomic data [17], α-amylase genes (17558, 22983) exhibited significantly higher expression during mid–late fermentation and maturation, most likely driven by starch demand, environmental changes, and microbial shifts, which emphasizes their critical role in starch liquefaction of NF Daqu. Conversely, α-glucosidase (15963) showed early-stage dominance in HB, stabilizing later, reflecting microbial community-driven enzyme regulation [15].

Temperature/pH profiling revealed distinct activity optima of saccharification, JXL (pH 5.0–7.0, 60–70 °C; peak at 21 days) vs. HB (pH 5.0, 65 °C; peak at 23 days), likely influenced by variations in the Daqu-making environment (temperature, humidity, handling practices, season, geography) [24,32,64,65,66,67] and the sensitivity of rare microbes to environmental changes [68]. Furthermore, the saccharification activities peaked at 21 days for JXL and 23 days for HB Daqu, coinciding with the transition from the high-temperature stage to the maturation stage, indicating enhanced hydrolytic activity at this phase [18].

Thermostable saccharification enzymes dominated mature NF Daqu samples from both the JXL and HB distilleries, which reflects their adaptation to high-temperature fermentation (~60 °C) that selects for heat-resistant microbes and enzymes [17,37]. Multiple activity peaks during fermentation indicated a dynamic accumulation of diverse starch hydrolases, implying the formation of a multifunctional enzymatic system in NF Daqu [17,69]. Despite their geographical proximity, JXL and HB exhibited distinct saccharificationactivity patterns: similar peak levels in mature Daqu but divergent succession trajectories. Initial low activity (<0.23 U/g) in both types likely arose from endogenous hydrolases present in raw wheat, e.g., α-amylase, glucoamylase and β-amylase [10,11,12,13], as the Daqu production process omits cooking and microbial growth is extremely slow at the beginning (0 d) [15,70].

Parallel analysis of α-glucosidase revealed stark inter-distillery contrasts. JXL showed activity peaking at the maturation stage (30 days, pH 7.0/40 °C), which suggests the gradual development of microbial communities during the maturation phase [18], whereas HB displayed negligible activity throughout fermentation, potentially due to microbial or procedural differences. Even at the maturation stage, HB’s α-glucosidase activity under identical conditions remained markedly lower than JXL’s, which underscores the existence of environment-dependent enzyme regulation. Integrating saccharification and α-glucosidase profiles, JXL demonstrated superior starch degradation capacity, which is consistent with its higher hydrolase gene expression. These findings highlight how localized production conditions shape enzymatic efficiency in NF Daqu ecosystems.

To validate the functional relevance of starch-hydrolyzing enzymes in NF Daqu, metagenomic analysis was performed on samples from the JXL and HB distilleries. Phylogenetic clustering revealed distinct branches for key hydrolase genes from GH15 (glucoamylase: 17723, 22243, and 19398), GH31 (α-glucosidase: 15963 and 31847), and GH13 (α-amylase: 22983 and 17558) families, confirming their functional specificity as sub-units within these enzyme groups [44,71,72,73,74]. Primers targeting these genes were capable of not only successfully detecting their presences in NF Daqu but also amplifying homologous sequences with similarities ranging from 38.7% to 100%, which confirmed their sub-functional distributions. The independent phylogenetic clustering of these enzymes, to some extent, indicates their distinct roles in starch degradation and underscores the functional complexity of the Daqu system. These findings elucidate inter-distillery variations in enzyme distribution, offering critical insights for optimizing Daqu quality evaluation.

Metagenomic analysis revealed GH13 as the most abundant starch hydrolase family throughout the Daqu-making process in both JXL and HB Daqu samples, followed by GH31 and GH15. This trend mirrors prior findings about the enzymatic profile of *Aspergillus nidulans* cultivated on cereal starches [75] and the prevalence of GH13 enzymes in the human gut microbiome [76]. HB exhibited higher GH13/GH15/GH31 gene counts than JXL, which suggests a more robust polysaccharide degradation network shaped by environmental and raw material factors.

JXL and HB Daqus employ divergent starch degradation strategies: JXL exhibits higher enzyme expression/activity, while HB maintains greater gene abundance, as a result of which it might be reasonable to hypothesize that different methods of post-transcriptional regulation or achieving enzyme stability for starch-degrading enzymes exist between HB and JXL Daqus. This functional dichotomy highlights distinct mechanisms of enzymatic adaptation to NF Daqu’s polysaccharide-rich environment, reflecting system-level optimization under varying production pressures [22,49]. Further experimental validation is needed to confirm these hypotheses.

GH13 genes persisted throughout the Daqu-making process, similar to the consistent presence of α-amylase gene 17558, which highlights the primary contributors of their enzyme members to starch degradation [18], particularly the 17558 enzyme (thermostable, GH13) [37]. Notably, these three hydrolase family genes were absent in the initial fermentation stage (0 day) of JXL Daqu, while GH13 family genes were detected in the 0-day HB Daqu, likely due to wheat-associated microbes. This discrepancy may stem from differences in wheat varieties and storage durations between the two distilleries [77]. GH31 family members were stably present in both the fermentation and maturation stages of HB Daqu, and only present in the maturation stage of JXL Daqu, with considerably lower gene abundance than in the HB Daqu. Strangely, GH15 enzymes appeared uniquely in the JXL Daqu at day 8, contrasting with previous DNA-level amplification, which is potentially due to the amplification of wheat-derived homologs [49] or incomplete annotations and unclassified sequences. Conversely, GH15 enzymes were consistently detected across all Daqu-making stages in HB Daqu, which aligns with DNA-based amplification. All these observations suggest that the distribution of starch-degrading enzymes in Daqu is closely linked to regional variations, environmental factors, and the raw material used [77].

α-glucosidase genes dominated both NF Daqus, followed by α-amylase and glucoamylase, closely aligning with the observed gene counts for the GH31, GH13, and GH15 families, respectively. The α-glucosidases were also previously reported as the most abundant enzymes during Daqu fermentation [18,49]. Their differential abundances reflect their sequential roles: α-amylase (endo-acting) breaks starch into dextrins and is processed by α-glucosidase/glucoamylase (exo-acting) into glucose/maltose [9]. Their synergy, particularly α-glucosidase’s efficiency in dextrin hydrolysis [78], underpins Daqu’s starch-degradation capacity.

Pearson analysis identified a positive correlation between expression levels of most detected genes and the saccharification activity, with gene_15963 (GH31) demonstrating the strongest positive correlation, which affirms its pivotal role in NF Daqu [79]. In contrast, glucoamylase gene_19398 (GH15) showed negative correlations, which indicates its limited contribution to or inhibition of the starch-degrading capacity of NF Daqu. Furthermore, gene_31847 (GH31), gene_15963 (GH31), and gene_22243 (GH15) also exhibited strong correlations with α-glucosidase activity, which indicates their regulation of this activity in NF Daqu, while α-amylase genes (17558, 22983) exhibited weaker links, aligning with their distinct catalytic mechanisms, i.e., the primary roles of the endo-acting (α-amylase) than the exo-acting (α-glucosidase) enzymes in degrading starch [9]. Additionally, the central role of α-glucosidase gene_15963 in starch-degrading activity—consistent with the broad specificity of its enzyme product on α-glycosidic bonds [44]—strengthens the overall findings of this study.

This study elucidates the intricate starch degradation dynamics in NF Daqu, which are governed by microbial community succession, enzymatic synergy, and region-specific production parameters [80]. Comparative analysis revealed distinct enzymatic adaptation strategies: JXL Daqu exhibited higher hydrolase activity compared to HB’s elevated gene abundance. The developed high-throughput primer design framework enables precise correlation of gene expression profiles with enzymatic kinetics, surpassing conventional PCR in data richness and genomic resolution for complex microbial systems. This methodological advancement provides actionable targets for Daqu optimization while complementing prior studies on glycoside hydrolase functionality [81], reinforcing the necessity of precise enzyme expression analysis for industrial process enhancement.

## 5. Conclusions

This study establishes an efficient molecular toolkit for quantitatively assessing the starch-degrading capacity of NF Daqu. By synergistically combining previous metatranscriptome analysis, DNA-level amplification, qPCR-based gene detection, and enzymatic activity profiling across both fermentation and mature stages, five core enzyme genes critical to starch hydrolysis were identified through Pearson correlation analysis. Furthermore, divergent starch degradation strategies were identified between JXL and HB Daqu: JXL Daqu exhibited higher enzyme expression and activity, while HB Daqu showed greater gene abundance. The developed primers and methodological framework elucidate the functional contributions of these enzymes within NF Daqu’s complex multi-enzyme system. These findings facilitate precision quality control by replacing empirical methods with RNA-/DNA-based biomarkers for standardized fermentation monitoring. They also support resource-optimized processing by targeted enzyme regulation to minimize raw material waste and enhance starch utilization. Additionally, strain-specific optimization can be achieved by harnessing either JXL’s high-activity or HB’s high-diversity strategies, which would promote sustainable Baijiu production.

Looking forward, future research could explore the ecological mechanisms driving the distinct starch degradation strategies observed in different Daqu types and investigate the integration of these molecular biomarkers into intelligent fermentation monitoring systems. This work bridges traditional brewing with data-driven food technology, providing actionable solutions for precision fermentation control and contributing to sustainable food systems, in alignment with Foods’ mission.

## Figures and Tables

**Table 1:** Specific and degenerate primers used in this study.

Primer	Forward and Reverse Sequence (5′→3′)	Amplicon Size (bp)	Annealing Temperature (°C)	Type of Primer	The Glycoside Hydrolase Family
GH15-1	F-CCTACTGGACGGGCTCGTAC	502	60	Specificity	GH15
	R-GGGGTGTATTTTTGCGCCAC				
GH15-4	F-AGCAATTCTCCCGCAACGAC	578	55		
	R-TCATCTCCAGCTATCGTTC				
GH13-1	F-CGGGTGGTGGTACAATGTCG	155	55		GH13
	R-ATGCGAATAGCAGTACTTTG				
GH13-3	F-CAATCGCTCGTGGCCAATTC	984	60		
	R-CATAATCGCTGCTACCCGCC				
GH31-1	F-CCATTGCCTGGATTCCC	310	56		GH31
	R-CGGCAAAAGTGCTTCGAG				
GH31-3	F-GATGTTGCCAAGCAGTACGC	651	57		
	R-CTGGAACACATCCGGGAATC				
22243	F-GASATCATCTGGCCGATTG	867	55	Degeneracy	GH15
	R-CYGRSGGAGTRTATTTCT				
19398	F-CVACGGCSMTGATTGCMT	1344	55		
	R-CRTCBGTCTCYTTYTTGA				
17723	F-GAYTTYATGTGCTGGCC	890	49		
	R-ACCCAKGARTARCGRTA				
22983	F-TGGATGCYGYBAARCAYT	560	55		GH13
	R-GTTGCCRTAKCGRACGAAG				
17558	F-GTATCACGGTTATTGGCAGAA	220	54		
	R-GAGACAGAACGGGTGGAAG				
31847	F-ATCTATGACSSBGATGAGG	697	56		GH31
	R-TRTCSGTCCACATKGTCTC				
15963	F-ATCCTCTGCCAACCCACT	823	56		
	R-GGCTCCAGCATAATACGAC				

**Table 2:** Primers for RT-qPCR.

Primer	Forward and Reverse Sequence (5′→3′)	Amplicon Size (bp)	Annealing Temperature (°C)
q15963	F-GGAACGCCAATCGTGAGA	197	56
	R-CCCTGGTAGACGGTGTAGTC		
q31847	F-CTTGAAAGATGCGGATGG	187	56
	R-AGAAGTTGGACGGCTCGT		
q22983	F-TAGCAAACCACGATACTCAA	228	54
	R-GCACTCCGTAGGCATAAA		
q17558	F-TACACCTGCCCATACCAGGA	198	60
	R-CGAAGCAAAGCGAGCAATGT		
q22243	F-CCTATGCGGACGGGTTTC	280	56
	R-GGTGGCTGCGTAGTGGTT		
q19398	F-AAGCGACCACAACCGCAACC	129	60
	R-TCGAGCCGACGAGGAAGACG		
q17723	F-CCTACAGGTGCCGTCGTT	193	56
	R-GTTGAGGCGGGAAGATGC		
*gapdh*	F-CGGCATCGTTGAGGGTCT	279	57
	R-GCCTTCTTGATCTCGTCGTA		

## Data Availability

The raw data from shotgun metagenomic sequencing, available in the NCBI SRA database (PRJNA1229847), will be provided upon request.

## Supplementary Materials

The following supporting information can be downloaded at: https://www.mdpi.com/article/10.3390/foods14183239/s1, Figure S1: Daqu samples were collected in the fermentation process of JXL and HB Daqu; Figure S2: Specific amplification results of DNA from different Daqu samples; Figure S3: PCR validation of specific primers for RT-qPCR in JXL and HB Daqus; Figure S4: Melting curves of test genes and reference genes in JXL and HB Daqus; Figure S5: Enzymatic characteristics of α-glucosidase during fermentation stages of JXL and HB Daqus; Figure S6: Phylogenetic tree of GH15 and GH31 family genes during fermentation and storage of JXL and HB Daqu; Figure S7: Phylogenetic tree of GH13 family genes during fermentation and storage; Table S1: Gene information of hydrolase family enzymes in the fermentation stage of Daqu-making process; Table S2: Expression levels of primer’s gene template; Table S3: Function prediction of template genes.

## Abbreviations

The following abbreviations are used in this manuscript:

NF	Nong-flavor
RT-qPCR	Reverse transcription quantitative PCR
GH	glycoside hydrolase
