# Supplementary material for: Spatiotemporal Profiling of Starch-Degrading Enzymes in Nong-Flavor Daqu: Molecular Markers for Quantitative Quality Evaluation
Source: Foods. 2025 Sep 18;14(18):3239. doi: 10.3390/foods14183239 (PMC12469301; doi:10.3390/foods14183239)
Supplement: Supplementary file 1 [file foods-14-03239-s001.zip › foods-3836687-supplementary.pdf]

Supplementary Material

Spatiotemporal Profiling of Starch-Degrading Enzymes in Nong-Flavor Daqu: Molecular Markers for Quantitative Quality Evaluation

Yijia Jiang [1,2,†], Yue Lu [1,2,†], Yanling Jin [1], Yi Shen [3], Nian Liu [4], Shu Bao [5], Kui Peng [4], Langfei Gan [3], Chaokai Wang [4], Yuling Zhang [1,2], Lanchai Chen [6], Bo Chen [3], Yao Xiao [7], Kaize He [1], Zhuolin Yi [1,*] and Hai Zhao [1,*]

[1] Agricultural Microbial Agents Key Laboratory of Sichuan Province, Chengdu Institute of Biology, Chinese Academy of Sciences, Chengdu 610213, China; jyj1122330818@163.com (Y.J.); oneluyue@163.com (Y.L.); jinyl@cib.ac.cn (Y.J.); 323086002124@stu.suse.edu.cn (Y.Z.); hekz@cib.ac.cn (K.H.)

[2] Liquor Brewing Biotechnology and Application Key Laboratory of Sichuan Province, Sichuan University of Science and Engineering, Yibin 644005, China

[3] Sichuan Langjiu Co., Ltd., Luzhou 646523, China; 15228262888@163.com (Y.S.); ganlf1898@163.com (L.G.); chenbo@langjiu.cn (B.C.)

[4] Sichuan Food and Fermentation Industry Research & Design Institute, Chengdu 611130, China; liunian999@163.com (N.L.); 1314520pk@163.com (K.P.); gswangchaokai@126.com (C.W.)

[5] Sichuan Liquor Research Institute Co., Ltd., Chengdu 610017, China; baoshujks@outlook.com

[6] School of Food and Bioengineering, Xihua University, Chengdu 610039, China; chenlc@xhu.edu.cn

[7] Analytical and Testing Center, Sichuan University of Science and Engineering, Zigong 643000, China; xiaoy828@126.com

[*] Correspondence: yizl@cib.ac.cn (Z.Y.); zhaohai@cib.ac.cn (H.Z.); Tel.: +86-28-82890725 (Z.Y. & H.Z.)

[†] These authors contributed equally to this work.

Table S1 Gene information of hydrolase family enzymes in the fermentation stage of Daqu-making process

The glycoside hydrolase family	Daqu fermentation stage	Gene numbers	Expression level (RPKM)
GH13	N2	35	973.2
	N3	36	556.8
	N4	10	47.0
GH31	N2	26	492.0
	N3	17	937.3
	N4	6	<0.5
GH15	N2	15	226.0
	N3	17	153.1
	N4	11	95.9

Table S2 Expression levels of primer's gene template

Primers	Gene ID	Expression level (RPKM)
GH15-1	N4_19398	83.4
	N4_19409	<0.5
GH15-4	N4_19398	83.4
	N4_11206	<0.5
	N3_39122	<0.5
GH13-1	N3_32956	<0.5
	N1_17266	125.2
GH13-3	N3_17558	293.5
	N3_35330	<0.5
GH31-1	N3_31847	655.5
	N3_31845	<0.5
GH31-3	N3_31845	<0.5
	N3_31847	655.5
22243	N3_22243	117.6
19398	N4_19398	459.8
17723	N3_17723	21.8
22983	N4_22983	10.6
17558	N3_17558	293.5
31847	N3_31847	655.5
15963	N3_15963	127.1

Table S3 Function prediction of template genes

Primers	Samples	Genes sources	Putative function	EnzymeSequence Similarity (%)	GenBank ID
GH15-1	N2	*Aspergillus steynii IBT 23096*	glucoamylase	46.9	XM_024846890.1
	N3	*Aspergillus oryzae*	glucoamylase	51.3	MH645916.1
	N4	*Paecilomyces variotii*	glucoamylase	60.1	XM_028632043.1
	JXL	*Paecilomyces variotii*	glucoamylase	42.5	XM_028632043.1
	HB	*Paecilomyces variotii*	glucoamylase	41.3	XM_028632043.1
	JLX	*Aspergillus melleus*	glucoamylase	47.9	XM_046091878.1
	RH	*Aspergillus melleus*	glucoamylase	44.7	XM_046091878.1
	RH 机	*Aspergillus melleus*	glucoamylase	44.7	XM_046091878.1
GH15-4	N3	*Paecilomyces variotii No. 5*	glucoamylase	71.7	BAUL01000135.1
	N4	*Aspergillusneoniger CBS 1156565*	α-amylase	48.0	XM_025621180.1
	JXL	*Paecilomyces variotii*	glucoamylase	73.3	XM_028632043.1
	HB	*Rasamsonia emersonii*	glucoamylase	66.4	AJ304803.1
	JLX	*Aspergillus lentulus*	glucoamylase	60.7	XM_033556905.1
	RH	*Aspergillus fischeri NRRL 181*	glucoamylase	62.5	XM_001265626.1
	RH 机	*Aspergillus fischeri NRRL 181*	glucoamylase	62.5	XM_001265626.1
GH13-1	N4	*Penicillium camemberti*	α-amylase	94.1	HG793152.1
	JXL	*Penicillium camemberti*	α-amylase	92.2	HG793152.1
	HB	*Penicillium camemberti*	α-amylase	94.1	HG793152.1
	JLX	*Penicillium camemberti*	α-amylase	94.1	HG793152.1
	RH	*Penicillium camemberti*	α-amylase	94.1	HG793152.1
	RH 机	*Penicillium camemberti*	α-amylase	94.1	HG793152.1
GH13-3	JXL	*Penicillium roqueforti*	α-amylase	59.0	KAI2720858.1
	HB	*Penicillium roqueforti FM164*	α-amylase	59.0	HG792016.1
	JLX	*Penicillium roqueforti*	α-amylase	58.9	XM_039078060.1
	RH	*Penicillium roqueforti*	α-amylase	59.0	XM_039078060.1
	RH 机	*Penicillium roqueforti*	α-amylase	58.0	XM_039078060.1
GH31-1	N3	*Thermomyces lanuginosus*	α-glucosidase	96.6	EU530574
	N4	*Thermomyces lanuginosus*	α-glucosidase	96.6	EU530574
	JXL	*Thermomyces lanuginosus*	α-glucosidase	97.5	EU530574
	RH	*Thermomyces lanuginosus*	α-glucosidase	74.7	EU530574
GH31-3	N3	*Thermomyces lanuginosus*	α-glucosidase	89.0	ACB13188.1
	N4	*Thermomyces lanuginosus*	α-glucosidase	90.0	ACB13188.1
22243	N3-1	*Thermomyces lanuginosus*	glucoamylase	98.5	AY948384.1
	N3-2	*Thermomyces lanuginosus*	glucoamylase	81.9	AY948384.1
	N3-3	*Thermomyces lanuginosus*	glucoamylase	81.6	AY948384.1
	N4-1	*Thermomyces lanuginosus*	glucoamylase	80.9	AY948384.1
	N4-2	*Thermomyces lanuginosus*	glucoamylase	81.6	AY948384.1
	N4-3	*Thermomyces lanuginosus*	glucoamylase	80.7	AY948384.1
	N4-4	*Thermomyces lanuginosus*	glucoamylase	98.9	AY948384.1
	JXL-1	*Thermomyces lanuginosus*	glucoamylase	81.9	AY948384.1

	JXL-2			78.6	
	JXL-3			81.5	
	JXL-4			87.4	
	HB-1	*Thermomyces lanuginosus*	glucoamylase	81.5	AY948384.1
	HB-2			80.3	
	HB-3			80.3	
	HB-4			79.2	
	JLX-1	*Thermomyces lanuginosus*	glucoamylase	81.9	AY948384.1
	JLX-2			81.9	
	JLX-3			82.2	
	RH-1	*Thermomyces lanuginosus*	glucoamylase	81.47	AY948384.1
	RH-2			98.2	
	RH-3			81.9	
	RH-4			98.9	
19398	N3-1	*Paecilomyces variotii*	glucoamylase	80.3	XM_028632043.1
	N3-2			80.3	
	N3-3			80.3	
	N3-4			80.3	
	JXL-1	*Paecilomyces variotii*	glucoamylase	79.0	XM_028632043.1
	JXL-2			80.3	
	HB-1	*Paecilomyces variotii*	glucoamylase	79.0	XM_028632043.1
	HB-2			78.5	
	JLX-1	*Paecilomyces variotii*	glucoamylase	80.3	XM_028632043.1
	JLX-2			80.3	
	RH-1	*Paecilomyces variotii*	glucoamylase	78.1	XM_028632043.1
	RH-2			80.3	
	RH-3			80.3	
17723	N3-1	*Paecilomyces variotii*	glucoamylase	75.3	XM_028633223.1
	N3-2			76.6	
	N3-3			76.6	
	N3-4			76.6	
	N4-1	*Paecilomyces variotii*	glucoamylase	76.6	XM_028633223.1
	N4-2			76.6	
	N4-3			39.0	
	N4-4	*Rasamsonia emersonii*		69.1	XM_013474261.1
	JXL-1	*Paecilomyces variotii*	glucoamylase	84.5	XM_028633223.1
	JXL-2			61.4	
	JXL-3			76.6	
	JXL-4			76.6	
	HB-1	*Paecilomyces variotii*	glucoamylase	76.6	XM_028633223.1
	HB-2			39.5	
	HB-3			75.1	
	HB-4			38.7	

	JLX-1	*Paecilomyces variotii*	glucoamylase	75.3	XM_028633223.1
	JLX-2			61.4	
	RH-1	*Paecilomyces variotii*	glucoamylase	76.6	XM_028633223.1
	RH-2			69.9	
	RH-3			76.6	
22983	N3-1	*uncultured fungus*	α-amylase	48.9	MT849765.1
	N3-2			98.7	
	N3-3			71.8	
	N3-4			70.4	
	N4-1	*uncultured fungus*	α-amylase	72.6	MT849765.1
	N4-2			70.4	
	N4-3			71.7	
	N4-4	*Aspergillus lentulus*		39.1	XM_033563982.1
	JXL-1	*uncultured fungus*	α-amylase	71.8	MT849765.1
	JXL-2			70.0	
	JXL-3			64.3	
	HB-1	*uncultured fungus*	α-amylase	70.4	MT849765.1
	HB-2			71.1	
	HB-3			95.5	
	JLX-1	*uncultured fungus*	α-amylase	71.1	MT849765.1
	JLX-2			62.7	
	JLX-3			63.4	
	JLX-4			71.1	
	RH-1	*uncultured fungus*	α-amylase	68.1	MT849765.1
	RH-2			71.1	
31847	N3-1	*Paecilomyces variotii*	α-glucosidase	80.0	XM_028626146.1
	N3-2	*Thermomyces lanuginosus*	α-glucosidase	100.0	EU530574.1
	N4-1	*Thermomyces lanuginosus*	α-glucosidase	99.5	EU530574.1
	N4-2			99.5	
	N4-3			99.5	
	N4-4			99.5	
	JXL-1	*Thermomyces lanuginosus*	α-glucosidase	99.5	EU530574.1
	JXL-2	*Paecilomyces variotii*	α-glucosidase	79.6	XM_028626146.1
	RH-1	*Thermomyces lanuginosus*	α-glucosidase	100.0	EU530574.1
	RH-2			94.5	
	RH-3			99.0	
	RH-4			99.5	
15963	N3-1	*Aspergillus flavus*	α-glucosidase	100.0	RAQ52401.1
	N3-2	*Aspergillus sergii*	glycosyl hydrolases	100.0	KAE8328942.1
	N4-1	*Aspergillus sergii*	glucoamylase	98.7	KAE8328942.1
	N4-2	*Aspergillus parasiticus*	glucoamylase	98.7	KAB8200528.1
	N4-3	*Aspergillus arachidicola*	α-glucosidase	98.7	PIG86804.1
	JXL-1	*Aspergillus flavus*	α-glucosidase	100.0	RAQ52401.1

JXL-2	*Aspergillus minisclerotigenes*	glycosyl hydrolases	100.0	KAB8274187.1
JLX-1	*Aspergillus parasiticus*	glycosyl hydrolases	100.0	KAB8200528.1
JLX-2	*Aspergillus arachidicola*	α-glucosidase	100.0	PIG86804.1
RH-1	*Aspergillus flavus*	α-glucosidase	100.0	RAQ52401.1
RH-2	*Aspergillus parasiticus*	glycosyl hydrolases	99.2	KAB8200528.1

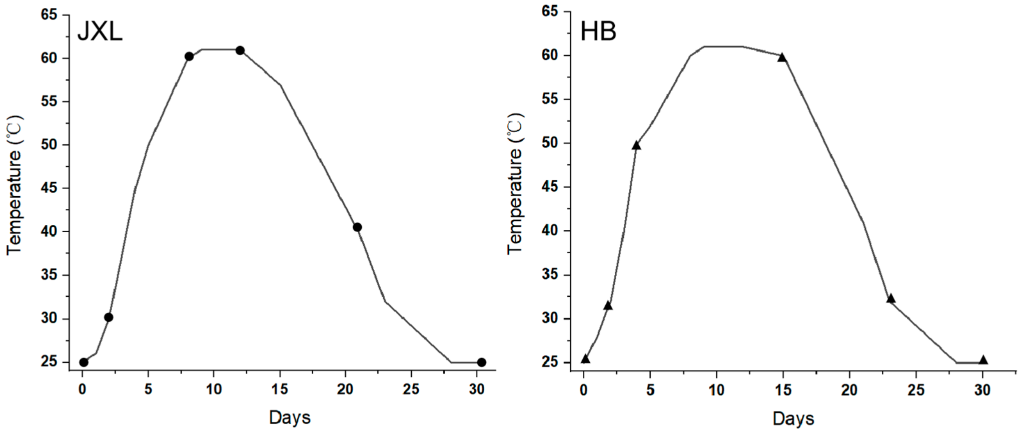

Figure S1 Daqu samples were collected in the fermentation process of JXL and HB Daqu. Samples were collected from fermentation stages of JXL Daqu (0, 3, 8, 12, 21, 30 days) at 25 °C, 30 °C, 60 °C, 61 °C, 42 °C and 25 °C, and HB Daqu (0, 2, 4, 15, 23, 30 days) at 25 °C, 32 °C, 50 °C, 60 °C, 32 °C and 25 °C, respectively.

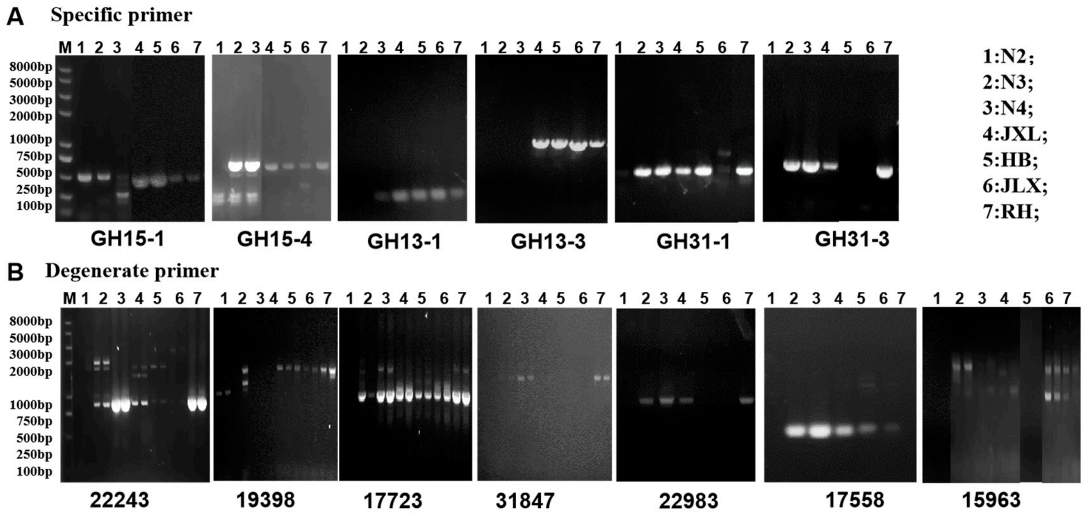

Figure S2 Specific amplification results of DNA from different Daqu samples

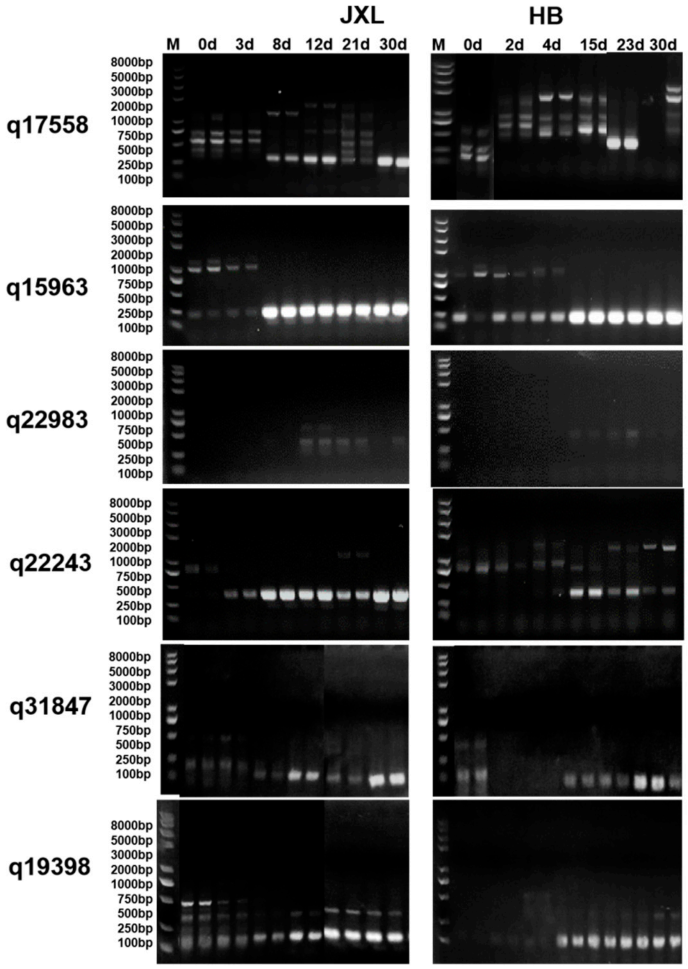

Figure S3 PCR validation of specific primers for RT-qPCR in JXL and HB Daqus

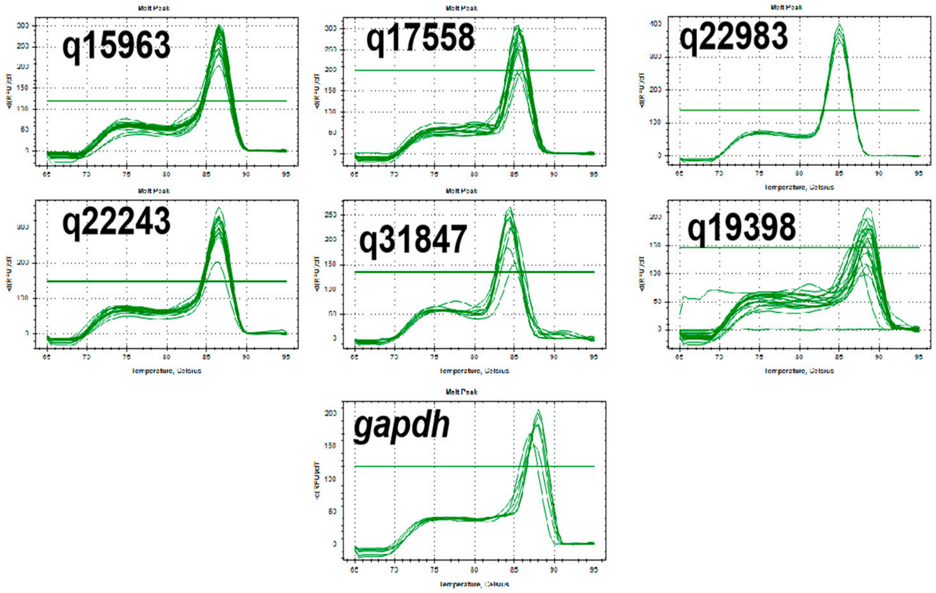

Figure S4 Melting curves of test genes and reference genes in JXL and HB Daqus

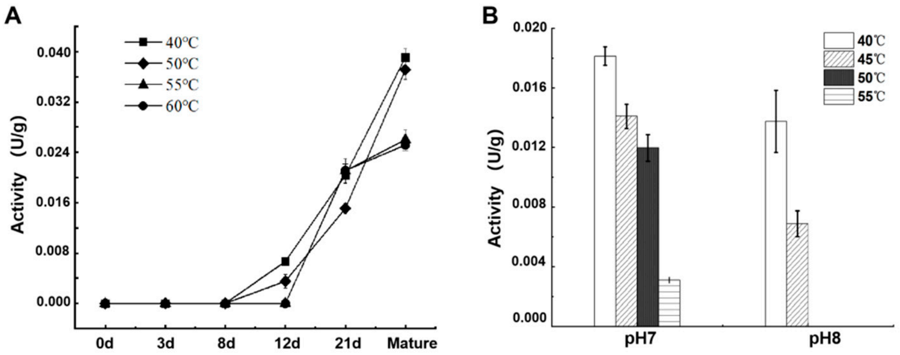

Figure S5. Enzymatic characteristics of α-glucosidase during fermentation stages of JXL and HB Daqus.

A: Alpha glucosidase activities at pH7 and different temperatures in fermentation process of JXL daqu;

B: Amylase activities at different temperatures and pHs in mature HB daqu.

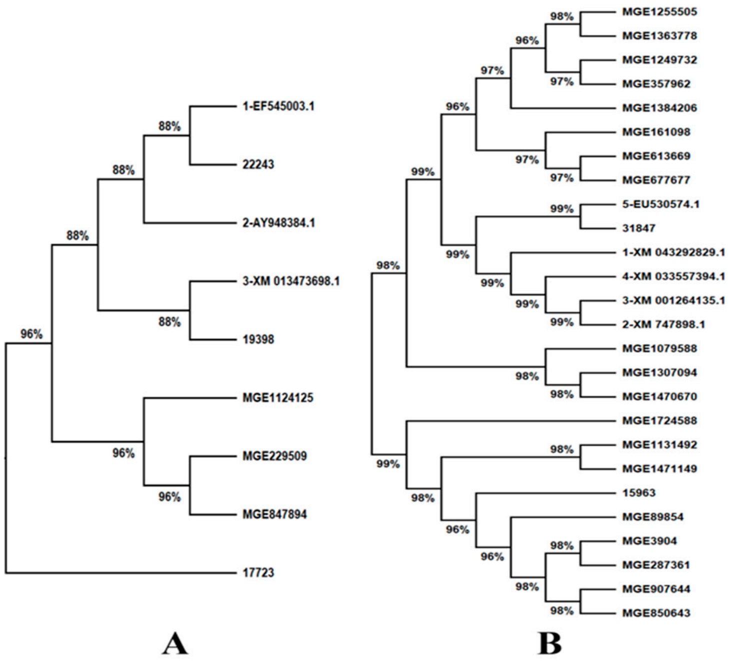

Figure S6 Phylogenetic tree of GH15 and GH31 family genes during fermentation and storage of JXL and HB Daqu.

A: GH15 family genes; B: GH31 family genes.

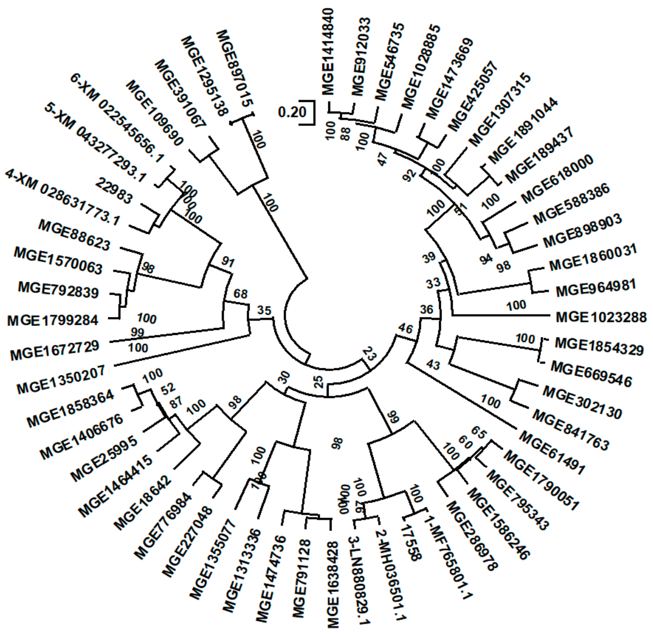

Figure S7 Phylogenetic tree of GH13 family genes during fermentation and storage
